# Role of Chondrocytes in Cartilage Formation, Progression of Osteoarthritis and Cartilage Regeneration

**DOI:** 10.3390/jdb3040177

**Published:** 2015-12-18

**Authors:** Hemanth Akkiraju, Anja Nohe

**Affiliations:** Biological Sciences, University of Delaware, Newark, DE 19716, USA

**Keywords:** stem cells, chondrocytes, articular cartilage, extracellular matrix, collagen, synovial, bone

## Abstract

Articular cartilage (AC) covers the diarthrodial joints and is responsible for the mechanical distribution of loads across the joints. The majority of its structure and function is controlled by chondrocytes that regulate Extracellular Matrix (ECM) turnover and maintain tissue homeostasis. Imbalance in their function leads to degenerative diseases like Osteoarthritis (OA). OA is characterized by cartilage degradation, osteophyte formation and stiffening of joints. Cartilage degeneration is a consequence of chondrocyte hypertrophy along with the expression of proteolytic enzymes. Matrix Metalloproteinases (MMPs) and A Disintegrin and Metalloproteinase with Thrombospondin Motifs (ADAMTS) are an example of these enzymes that degrade the ECM. Signaling cascades involved in limb patterning and cartilage repair play a role in OA progression. However, the regulation of these remains to be elucidated. Further the role of stem cells and mature chondrocytes in OA progression is unclear. The progress in cell based therapies that utilize Mesenchymal Stem Cell (MSC) infusion for cartilage repair may lead to new therapeutics in the long term. However, many questions are unanswered such as the efficacy of MSCs usage in therapy. This review focuses on the role of chondrocytes in cartilage formation and the progression of OA. Moreover, it summarizes possible alternative therapeutic approaches using MSC infusion for cartilage restoration.

## 1. Introduction

Articular cartilage (AC) is a smooth viscoelastic tissue designed to bear and distribute loads across the diarthrodial joints. This highly specialized tissue exhibits a unique mechanical behavior and poor regenerative capacities. AC has an organized layered structure that is divided into four zones: superficial, middle, deep zone and the zone of calcified cartilage [1]. Chondrocytes in the AC proliferate and secrete extracellular matrix to maintain and sustain the cartilage. The cells themselves are separated from each other by cartilage matrix [2]. They respond to outside stimuli and tissue damage, and are also responsible for degenerative conditions, such as osteoarthritis (OA). There are no known drugs today that can retard or reverse the progression of OA. Recent research identified the Mesenchymal Stem Cell (MSC) niche in cartilage. This discovery led to advancements in cell based therapies for cartilage restoration, and may pave the way for future therapeutic interventions [3]. More research is necessary to understand these mechanisms in more detail. In order to understand the role of chondrocytes in cartilage repair and in the progression of OA, we will first need to understand their primary function within the cartilage.

## 2. Chondrocyte Function and Regulation

Chondrocytes of the AC perform different functions compared to chondrocytes of the epiphyseal growth plates. Chondrocytes of the AC aid in joint articulation, while chondrocytes of the growth plate regulate the growth of the epiphyseal plates. Since this review focuses on OA, we will relate only to chondrocytes of the AC. Chondrocytes are metabolically active cells that synthesize and turnover a large volume of extra cellular matrix (ECM) components such as collagen, glycoproteins, proteoglycans, and hyaluronan [2]. The metabolic activities of chondrocytes are altered by many factors that are present within their chemical and mechanical environment. Most important among these factors are the pro-inflammatory cytokines and growth factors that have anabolic and catabolic effects. These factors play a role in the degradation and synthesis of matrix macromolecules [4–6]. However, little is known about the molecular mechanism by which these growth factors and peptides elicit their effects on ECM metabolism. Chondrocytes are derived from MSCs and occupy only 1%–5% of the total cartilage tissue [7]. This low density is due to the high matrix to cell volume ratio [7,8]. Furthermore, the life span of the chondrocyte is controlled by the areas of its residence. Since AC is an avascular tissue, chondrocytes rely on diffusion of nutrients and metabolites from the articular surface [8]. Moreover, these cells function in a low oxygen environment with low metabolic turnover. They inherently contain low mitochondrial numbers [2]. The mechanosensitive chondrocytes are major contributors for ECM production and they provide the functional and mechanical ability to withstand compressional, tensile, and shear forces across the diarthrodial joints.

## 3. ECM Production and Its Regulation by Chondrocytes

**Figure 1:**
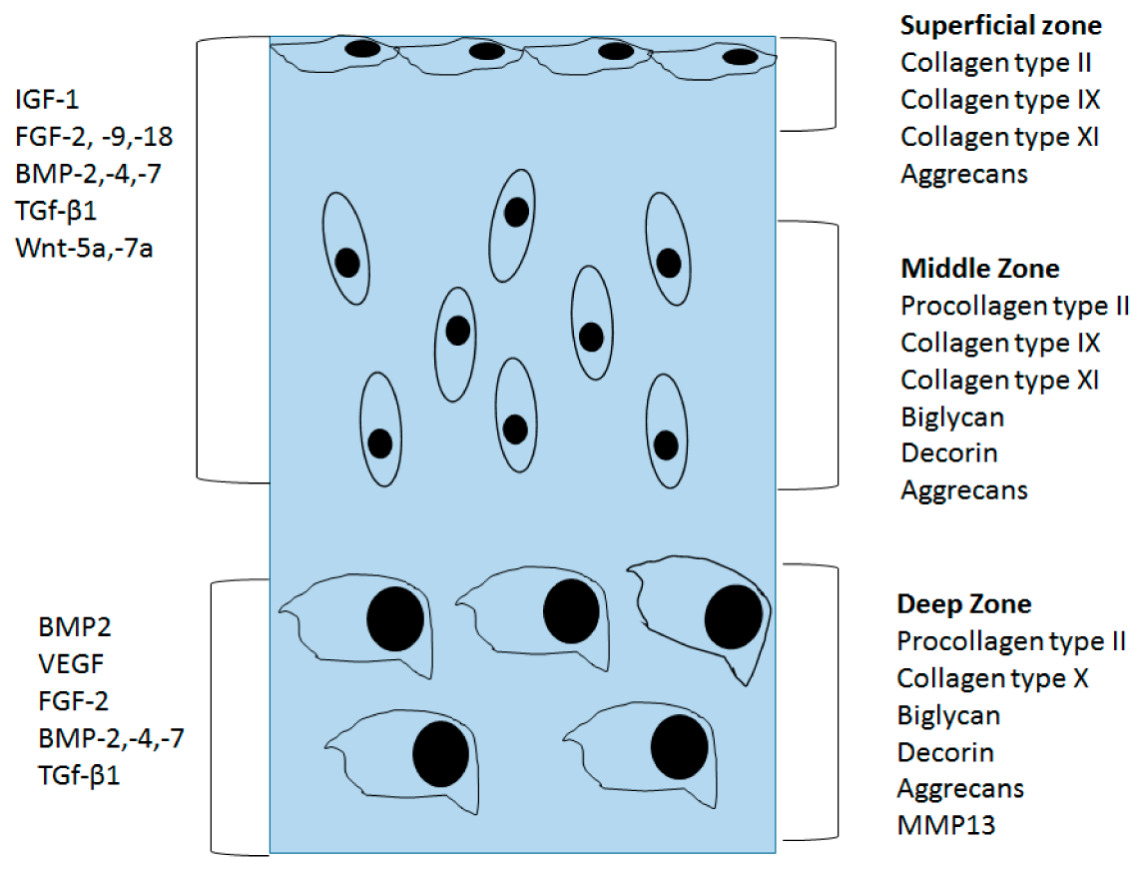
Organization of normal articular cartilage. Superficial, middle, and deep zones and their extracellular matrix is divided using different sections. Growth factors that control the chondrocyte function are divided based on the stage of chondrocyte lineage.

The main function of chondrocytes in the superficial and mid zone is to synthesize ECM composed of collagen type II, IX, and XI and proteoglycans. This ECM facilitates compressional and tensile forces across the diarthrodial joint [9,10]. Collagens are the most abundant macromolecules of the ECM, make up 60% of the dry weight of the cartilage, and provide tensile and shear strength to the tissue. Collagen also stabilizes the matrix. Collagen type II makes up 90%–95% of the collagen in ECM and forms fibrils and fibers interwoven with proteoglycan like aggrecans [10]. Collagens type IX and XI represent 5%–10% of the AC collagenous network and offer support for the collagen fibrilar crosslinking. Chondrocytes of the deep zone are terminally differentiated and actively synthesize collagen type X. Proteoglycans represent the second largest group of macromolecules and are heavily glycosylated protein monomers that resist compressional forces by swelling pressure due to their affinity to water across the articular joint [9]. These proteoglycans include aggrecan, decorin, biglycan, and fibromodulin. Aggrecans are the largest among the group [9,11]. Growth factors play a crucial role in controlling chondrogenesis by affecting MSCs differentiation to chondrocytes. They also influence chondrocytes to synthesize specific ECM proteins (Figure 1). The shift in expression of collagen type X by the chondrocytes also marks the regulation of proteolytic enzymes production. These enzymes aid in the clearing of the cartilage ECM and allow vascularization and calcification of tissue [10,12].

In order to maintain the homeostasis of the ECM, the synthesis and degradation of the ECM must be fine-tuned. Damage to AC tissue leads to loss of its ECM, followed by chondrocytes secreting new ECM to repair the damage. Although, chondrocytes are the primary contributors of AC ECM secretion, their turnover rates are not balanced. Proteoglycan turnover is estimated to take up to 25 years, while collagen half-life is estimated to range from several decades to 400 years [9,13]. Therefore, damages to the tissue can further play a role in the progression of slow degeneration of the tissue and elevate OA like conditions.

The composition of the ECM as well as the organization of chondrocytes and their response to external factors such as cytokines is dependent on the age of the tissue, however chondrocyte numbers remain unchanged [14]. In the course of aging, dissipation of chondrocytes in the superficial region is followed by an increase in the number of chondrocytes in the deep layers. Consequently the decrease in the hydration of the matrix results in an increased compressive stiffness. The age related decrease in the proteoglycan aggregate numbers within the ECM may be a result of proteolytic damage to the link proteins and glycosaminoglycan chains and increase in partially degraded hyaluronan without newly synthesized molecules [15–17]. Thus, increased mechanical forces exerted on the tissue further lead to subchondral tissue calcification [18,19]. These overall structural changes seen in the aging cartilage may just be another factor for the development of diseases, such as OA.

## 4. Structural Changes in OA Cartilage

OA is the most prevalent type of cartilage degenerative disease, the other being rheumatoid arthritis. OA results in progressive cartilage degradation characterized by the softening, fibrillation and erosions of the articular surface [20]. Breakdown of proteoglycans leads to a reduction in the compressive stiffness of the tissue that accelerates the rate of collagen loss [21]. In OA, besides cartilage erosion in subchondral bone, synovial fluid, and the synovial membrane also play a role in the progression of OA. Osteophyte formations, subchondral bone remodeling, and synovial membrane inflammation may further aid in cartilage tissue degradation. In early stages of OA, hypertrophic chondrocytes express collagen type X. This production marks the terminal differentiation of chondrocytes that regulates the expression of proteolytic enzymes like MMPs, and ADAMTS that degrade the proteoglycan and collagen network. Simultaneously, activation of transcriptional regulators such as Runt-Related Transcription Factor 2 (RUNX2) are known to induce terminal differentiation and enhance the expression of collagen type X and proteolytic enzymes that digest the AC ECM [22–24]. MMP-1 (Collagenase-1) and MMP-13 (Collagenase-3) are the primary factors that lead to overall degradation of collagenous framework. MMP-3 (Stromelysin-1) and ADAMTS-4 (aggrecanase-1) degrade proteoglycans [25,26]. It is shown that MMP activities are controlled by physiologic activators such as cathepsin B and tissue inhibitors of MMPs (TIMPs) [27]. An imbalance between these factors is commonly seen in OA tissue. Repeated mechanical insult to AC enhances MMP production and enhances cartilage matrix breakdown, [28,29]. These deleterious effects are pronounced in the superficial region of AC [30]. However, the process that regulates the production of proteolytic enzymes still remains unclear [30].

Inflammatory cytokines, such as IL-1β, TNF-α, and IL-6, are known to be upregulated during OA progression [4]. These inflammatory cytokines are secreted by chondrocytes and synoviocytes. They play a role in the disruption of cartilage homeostasis, and MMP mediated cartilage degradation [4,31] modulating the chondrocyte metabolism by increasing MMP expression and inhibiting the production of MMP inhibitors [27]. IL-1β mediated TNF-α expression has been shown to regulate IL-6 production and nuclear factor-κβ (NF-κβ) dependent transcriptional expression of Hypoxia-inducible factor 2α (HIF-2α) drive the processes that may further enhance AC destruction [32–37]. OA induced cartilage damage follows a myriad of cascades that once activated result in an irreversible damage to the tissue. Chondrocytes recognize the loss of ECM and actively produce collagen type II and proteoglycans. However, the ratio between the ECM protein production to proteolytic enzyme production is imbalanced and results in complete loss of cartilaginous tissue overtime. Moreover, cellular attempts to repair the tissue results in aberrant osteoblast like differentiation forming osteophytes or fibroblastic differentiation inducing fibrosis or stiffening of the joints [38–40].

## 5. OA Induced Osteophyte Formation and Fibrosis

OA pathology shows chondro/osteophyte formation and sclerosis of subchondral bone [39]. In addition synovitis is a common occurrence in OA, which involves osteophyte formation at the junction of periosteum and synovium [20,41–43]. Commonly osteophyte development is caused by MSCs near the periosteum as a form of repair mechanism to help stabilize the joints [20,41]. The increase in endogenous MSCs recruitment and chondrogenic differentiation in the damaged cartilage can be seen as a form of tissue repair and regeneration [44]. However, in this process aberrant expression of growth factors, such as Transforming Growth Factor β (TGF-β), BMP-2 and upregulation of other inflammatory responses, might leads to chondrocyte hypertrophy/apoptosis and osteophyte formation [45,46]. The mechanisms that control this activity remain unknown. Macrophages from the synovial lining enhance the inflammatory response and the cartilage damage. These synovial macrophages induce both anabolic and catabolic processes. Macrophages initiate these processes by secreting growth factors such as TGF-β, and BMP-2 [47,48].

The development of osteophytes causes negative effects, such as pain and loss of movement. The osteophytes are composed of cells expressing procollagen type 1 and type IIA [38,41,49]. Simultaneously, the production of spontaneous nitric oxide by chondrocytes and chondrocyte death allows osteophyte formation [38].

Another important hallmark of OA along with cartilage degeneration, and osteophyte formation is fibrosis that results in joint pain and stiffness. It results from the imbalance induced by the growth factor activity regulating matrix synthesis and degradation. Two main factors contribute to this, TGF-β and Connective Tissue Growth Factor (CTGF) [50,51]. Fibrosis results in fibrin deposition within the synovium. It causes joint stiffness that is another symptom in the progression of OA in combination with osteophyte formation and degradation of the AC. The body’s attempts at cellular repair include the recruitment of chondroprogenitors from the surrounding MSC niche. However, MSCs ability for multi lineage differentiation makes this an arduous process. Moreover, MSC differentiation relies on the signaling factors that control the cell turnover. Signaling in OA cartilage may be a potential problem for treatments in the long term.

## 6. Signaling in OA Cartilage

AC development, growth, maintenance, and repair are controlled by several signaling factors that trigger multiple bioactive roles within the chondrocyte metabolism. The regulatory mechanisms of the growth factors are responsible for cartilage homeostasis. An imbalance between them is often noticed in OA cartilage. The activated signaling cascades involved in OA progression are TGF-β1, BMP2/4/7, Wnt5a, Insulin Growth Factor 1 (IGF-1), and Fibroblast Growth Factor 2 (FGF-2) [22,52–54]. TGF-β1 together with BMP2/4/7, and IGF-1, contributes to cartilage formation and their mechanisms are extensively studied [5,55]. However, these anabolic growth factors are also catabolic in OA [24,56–58]. Similarly, HIF, NF-κβ pathway, Mitogen-Activation Protein Kinase (MAPK) pathways may contribute to OA progression [34,59].

**Figure 2:**
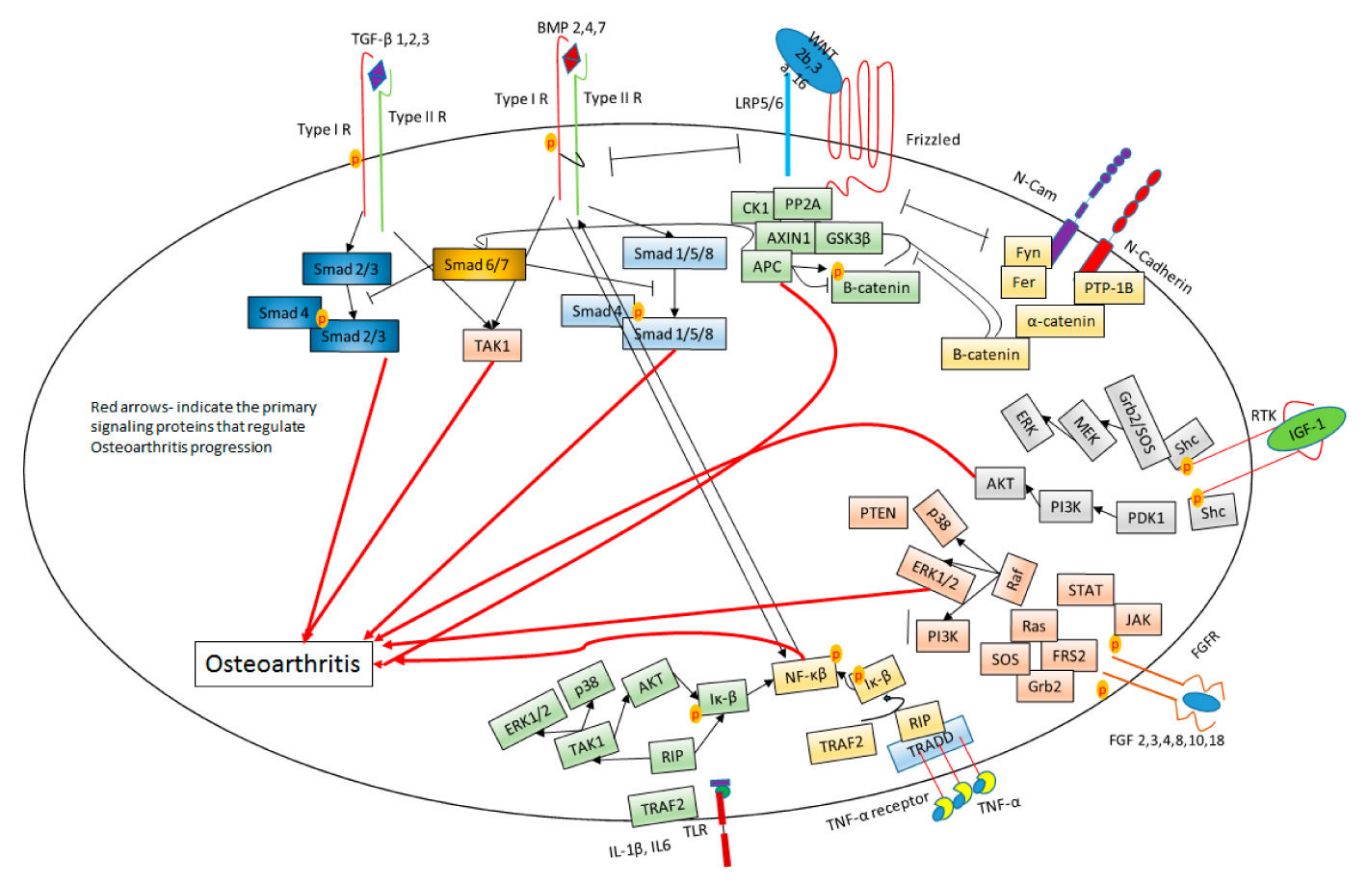
Signaling cascades involved in Osteoarthritis. Red arrows indicate the primary signaling protein that regulate OA progression. The black arrows signify the activation of the proteins. The bars indicate inhibition of the proteins.

The effect of BMPs on chondrogenesis was demonstrated. BMPs function promote differentiation, proliferation, and maturation throughout the chondrocytes lineage [60]. While BMP7 enhanced chondrogenic activity, BMP2 also induces chondrocyte hypertrophy. This is remarkable since both factors signal through the same receptors. The BMP canonical Smad 1/5/8 pathway is a potent inducer of chondrocyte hypertrophy and endochondral ossification [61]. Therefore, other pathways within BMP signaling may be responsible for the diversity of effects. During OA, BMP2 mRNA levels are upregulated and followed by terminal differentiation of chondrocytes [62]. The terminal differentiation of chondrocytes enhances the secretion of collagen type X and MMP-13. During progression of OA, several chondrocytes within the cartilage tissue express BMPs. Enhanced BMP production may influence the MSCs present in the OA cartilage. BMPs may potentiate chondrogenic differentiation but may also initiate aberrant osteophyte formation as well as enhance proteolytic enzyme production for the acceleration of cartilage degradation [63]. Crosstalk between BMP, TGF-β and Wnt signaling pathways is known to regulate terminal differentiation of chondrocytes and the differential modulation between these signaling pathways could accelerate OA [64,65]. Wnt-16, Wnt-2B, and Wnt-induced signaling protein 1 (WISP-1) are expressed at high levels in OA, similar to the level of BMPs [66]. BMP2-induced Wnt/β-catenin signaling enhances the low-density-lipoprotein receptor-related protein 5 catabolic activity, followed by promoting hypertrophy in osteoarthritic chondrocytes.[57]. Wnt/β-catenin negatively regulate NF-κβ and drive TGF-β/BMP signaling. This leads to enhanced expression of RUNX2 that enhances the expression of MMP-13, MMP3, and collagen type X [53]. This process drives chondrocyte hypertrophy and accelerates OA induced cartilage damage. Figure 2 summarizes the pathways involved in OA progression. How or what causes these imbalances in these signaling cascades is not known. Moreover, the regulation of the crosstalk between the factors is not completely understood.

## 7. Mesenchymal Stem Cell Niche for Cartilage Repair

During skeletal development chondrogenesis begins with mesenchymal cell recruitment, migration, and proliferation [58,67]. Condensation or aggregation of chondroprogenitor mesenchymal cells by cell–cell and cell–matrix interactions are associated with an increase in cell adhesion. This process can be measured by determining the levels of neural cadherin (N-cadherin), and neural cell adhesion molecule (N-CAM) [68]. Multiple growth factors, such as TGF-β, BMPs, FGFs, and Wnts, control the limb patterning for the development of AC of the epiphyseal plates and endochondral ossification of the metaphyseal plates [69].

A typical injury to tissues results in multiphase wound healing. It involves different phases in the order of inflammation, proliferation, and maturation. Any damage to the cartilage may go unrepaired and result in post-traumatic OA progression. Chondrocyte cell senescence may be another factor contributing to improper healing of subsurface cartilage injuries in addition to the lack of progenitor cells surrounding the tissue area [70]. Furthermore, the dense pericellular matrix (chondrons) surrounding the chondrocytes makes migration a challenging process. Cartilagenous ECM is constantly under pressure due to the swelling nature of the tissue and high tensile collagen network reinforcing it. This makes it even harder for chondrocytes to achieve the cellular motion within the tissue [71–73]. Although this process was identified *in vitro*, it is still difficult to gauge the chondrocytes capacity to migrate to the site of injury *in vivo* [73]. In a study by Kouri *et al*, OA tissue of fibrillar and non-fibrillar regions exhibited cell clustering effect. The cells proliferated and clustered in the regions of damage [74]. The study also demonstrated changes in the cytoskeletal arrangement by the presence of abundant filopodia and primary cilium. These data suggest the possibility of active movement of chondrocytes to areas of damage. Moreover, a recent study suggests that chondrocytes or chondroprogenitors migrate to the site of injury and repair the injury by synthesizing the lost ECM [73]. For this movement cells may remove the surrounding ECM by expressing proteolytic enzymes, and utilizing amoeboid locomotion [73]. Another study describes the differentiation and recruitment of chondroprogenitors through the synovial mesenchymal stem cell niche for cartilage repair [75,76]. Synovial cells plated on BMP coated plates differentiated into chondrocytes [77]. This suggests the influence of growth factors such as TGF-β/BMPs on synovial cells. These factors may induce the differentiation and migration of synovial stem cells to AC as an attempt to repair damaged cartilage tissue in OA [78]. Moreover, autologous synovial fluid was utilized to expand MSCs in tissue culture of synovium from OA patients [78]. There is also evidence that a progenitor cell population resides in the regions of synovial cavities, perichondrial Groove of Ranvier and in the infrapatellar fat pad [79–81]. Researchers demonstrated the presence of the known stem cell markers Stro-1, and Jagged-1 in the perichondrial Groove of Ranvier and also Stro-1, and BMPRIa in significant portion of the superficial zone of AC in three-month-old New Zealand white rabbits [79]. Furthermore, isolated stem cells from the infrapatellar fat pads and from the synovium regions demonstrated superior chondrogenic potential compared to that of mesenchymal stem cells derived from the bone marrow tissue [82,83]. Interestingly, cell populations that are expressing the stem cell markers such as Notch-1, Stro-1, and VCAM-1 were found to have increased expression in the superficial zone of OA cartilage than compared to the middle or the deep zone of AC [84]. These findings suggest the contribution of endogenous progenitors in synovium and infrapatellar fat pads for the renewal of AC.

## 8. Mesenchymal Stem Cell Therapy for OA Cartilage Repair

Current research aims to utilize cell-based therapies to reverse cartilage loss. These MSCs are isolated from bone marrow, adipose tissue, placenta, and umbilical cord. The ability of these MSCs to form cartilage is under rigorous investigation [85]. No specific markers have been identified for detecting MSCs populations. However, the International Society of Cell Therapy along with researchers have defined a few markers to distinguish stromal cells (CD73, CD105, CD109, *etc*.) from hematopoietic stem cell (CD45, Cd34, CD14, Cd19, CD11b, HLADR, *etc*.) (Table 1) [3,86,87]. Without a proper marker to identify the MSC populations it is difficult to study the biological properties of these cells. Although, bone marrow stromal cells (BMSCs) are known to differentiate into chondrocytes, adipocytes, and osteocytes controlling their fate of differentiation is a feat on its own [88,89]. Several models have shown that the quality of cartilage produced by BMSCs is equivalent compared to that using chondrocytes [90]. Other research uses scaffolds from several biomaterials, such as poly-lactic-co-glycolic acid sponge or fibrin gels, along with TGF-β1 and BMSCs [91]. These biomaterials demonstrated satisfactory cartilage tissue restoration among various other synthetic and natural scaffolds used for cartilage repair treatment [92]. Therefore, appropriate biomaterials and scaffolds may be necessary for the controlled differentiation of BMSCs for cartilage restoration. Several factors have to be considered for the treatment of BMSCs for the cartilage restoration. These include unwanted differentiation of cell fate influenced by the environmental factors that are present in persisting pathological condition. In practice, implants are still expensive, labor intensive and the transplants need to be cell source transplants that need to be placed in a suboptimal environment. Placing the implant in a wound or scar may provoke innate immunity that can hamper cell survival [93]. Fine tuning of this process requires a greater knowledge of the environmental influence on the cells of the patellar cavity and in AC. Therefore, it is important to study the long term effects of the cell based therapies in patients suffering from OA.

## 9. Conclusions

OA pathophysiology features loss of AC through the loss of cartilaginous ECM and the cells that are embedded in it. Chondrocytes are relatively inert cells with insufficient regenerative capacity. Overexpression of proteolytic enzymes including MMPs, ADAMTS further degrade the diseased cartilage. Common practice for mild OA treatments include using physiotherapy, and pharmacologic agents to reduce pain and inflammation. As the disease progresses intra-articular steroids or hyaluronic acid administration is the common practice [100]. However, while the patients experience temporary relief, its short lived and its effectiveness is debatable. In advanced cases of OA progression knee replacements are common [87,101].

Although, the causes for the degenerative disorders like OA are still unknown. MSC niche identification allowed us to understand the interactions between damaged cartilage and synovium. It also helps to identify their potential role in cartilage damage repair. Similarly, how the signaling cascades of TGFβ, BMP, Wnts, and FGF help in cartilage formation should be determined and utilized to retard or reverse the progression of OA. Cell-based MSC therapies for cartilage restoration made noteworthy progress. Regenerating a significant portion of the cartilage in OA alleviates pain. However, this process has not yet been studied in long-term cases. Moreover, cartilage formed by artificial MSC grafts has not been studied in detail for its mechanical properties. It may be a possible that OA-like conditions may resurface in the long term. While this process has made progress for treating OA patients, it is still unclear how these MSC graft therapy compare to the native cartilage in terms of its structure and functional longevity. There are still many questions to be answered. There are no drugs today that can stop or reverse the process. Therefore, a great deal of work is still needed to understand this degenerative disease, and for the development of therapeutics to stop the progression of OA.

## Figures and Tables

**Table 1:** Isolation potential of adult stem cell sources depending on the site specific areas of the patellar cavity.

Tissue Specific Progenitor Cells				
Tissue Source	Pros	Cons	Tissue Specific Cell Surface Markers	References
Bone Marrow	Can differentiate in to adipocytes, osteoblasts, and chondrocytes. High chondroprogenitor populations. High expansion potential	Heterogeneous population of cells. Invasive procedure to harvest	CD105, CD73, CD45, CD109, CD44, CD90, CD271, CD14, CD19, CD11b, CD13, CD166, CD146, CD34, SSEA-4, and HLADR	[3,86,87,94]
Synovium/synovial Fluid	Superior chondrogenic potential., regardless of donor age. High expansion potential	Heterogeneous populations of cells	CD105, CD73, CD44, CD90, CD271, CD13, CD166	[3,95–97]
Infrapatellar Fat Pad	Superior source of progenitor cells for chondrogenic differentiation potential	Early senescence, differentiation capacity to other tissues	CD105, CD73, CD44, CD166, D271, CD13, CD90, CD34, CD45, CD31, VEGF-2	[3,95,98,99]
